# High-throughput soybean seeds phenotyping with convolutional neural networks and transfer learning

**DOI:** 10.1186/s13007-021-00749-y

**Published:** 2021-05-05

**Authors:** Si Yang, Lihua Zheng, Peng He, Tingting Wu, Shi Sun, Minjuan Wang

**Affiliations:** 1 College of Information and Electrical Engineering, China Agricultural University, Beijing, 100083 China; 2 Institute of Crop Sciences, Chinese Academy of Agricultural Sciences, Beijing, 100081 China; 3 Key Laboratory of Modern Precision Agriculture System Integration Research, Ministry of Education, China Agricultural University, Beijing, 100083 China; 4 College of Information Science and Engineering, Shandong Agriculture and Engineering University, Jinan, 251100 China; 5 Key Laboratory of Agricultural Informatization Standardization, Ministry of Agriculture and Rural Affairs, China Agricultural University, Beijing, 100083 China; 6 College of Information Engineering, Northwest A&F University, Yangling, 712100 China

**Keywords:** Seed phenotyping, High throughput, Instance segmentation, Deep learning, Mask R-CNN

## Abstract

**Background:**

Effective soybean seed phenotyping demands large-scale accurate quantities of morphological parameters. The traditional manual acquisition of soybean seed morphological phenotype information is error-prone, and time-consuming, which is not feasible for large-scale collection. The segmentation of individual soybean seed is the prerequisite step for obtaining phenotypic traits such as seed length and seed width. Nevertheless, traditional image-based methods for obtaining high-throughput soybean seed phenotype are not robust and practical. Although deep learning-based algorithms can achieve accurate training and strong generalization capabilities, it requires a large amount of ground truth data which is often the limitation step.

**Results:**

We showed a novel synthetic image generation and augmentation method based on domain randomization. We synthesized a plenty of labeled image dataset automatedly by our method to train instance segmentation network for high throughput soybean seeds segmentation. It can pronouncedly decrease the cost of manual annotation and facilitate the preparation of training dataset. And the convolutional neural network can be purely trained by our synthetic image dataset to achieve a good performance. In the process of training Mask R-CNN, we proposed a transfer learning method which can reduce the computing costs significantly by finetuning the pre-trained model weights. We demonstrated the robustness and generalization ability of our method by analyzing the result of synthetic test datasets with different resolution and the real-world soybean seeds test dataset.

**Conclusion:**

The experimental results show that the proposed method realized the effective segmentation of individual soybean seed and the efficient calculation of the morphological parameters of each seed and it is practical to use this approach for high-throughput objects instance segmentation and high-throughput seeds phenotyping.

## Background

The legume species soybean (*Glycine max* L.), ranking among the top five worldwide major crops [1], is one of the most important grain legumes. Also, it is an important source of vegetable oil and protein for human consumption [2]. Crop yield of soybean highly depends on three major aspects which are the number of pods per plant, the number of seeds per pod and the seed size [3]. The size of soybean seed, which is not only a very important appearance quality but also strongly associated with the commercial value [4], is an important agronomic trait that affects the quality and yield of soybean [5]. The seed morphological phenotypes, which include seed shape, seed length, seed width, seed height, seed circumference, seed surface area and seed volume and so on, are essential to reflect the growth and development, physiology, biochemistry and genetics of soybean [6]. Paying attention to the morphological traits of soybean seeds is a powerful indicator for improving crop yield. However, Effective soybean seed phenotyping requires large-scale accurate quantities of accurate morphological phenotype parameters. Thus, it is necessary to develop an automatic approach to acquire accurate soybean seeds morphological parameters information.

The traditional acquisition method of soybean seed morphological phenotype relies on manual approaches by measuring and evaluating the shape of the seed with a vernier caliper. Since the size of soybean seeds is small generally, the operation of manual measurements is labor-intensive, time-consuming and error prone extremely. Moreover, the phenotypic information of manual measurement is limited to the seed length, seed width, and seed height, and no more information can be measured, it is not applicable for large-scale collection of soybean seeds morphological phenotype information [6].

With the rapid development of imaging technology, it is possible to measure the morphological phenotype information of high throughput soybean seeds, however the seeds need to be sparsely placed without physical contact [6]. Traditional image-based researches on soybean seed phenotyping mainly include seed quality evaluation [5, 7–9], seed counting [10–12], etc. Also, image-based quantification of seed morphological phenotype information is widely used in rice grain [13], corn grain [14], etc. Widely used open-source image analysis software for seed morphological phenotype quantification include SmartGrain [15], ImageJ [16], CellProfiler [17], P-TRAP [18] and WinSeedle [19] and so on. These software are mainly based on classic but ordinary image processing techniques to separate individual seeds, such as morphological open operation [15], watershed algorithm [16, 17], and handcrafted feature based bespoke algorithm [19], etc. Some scholars also proposed a traditional image processing technology based method to extract high-throughput soybean seeds phenotype information automatically [6]. These software and methods mentioned above can realize the phenotype parameters measurement of high throughput seeds which are sparsely placed without overlap under consistent light condition to achieve an effective segmentation. When soybean seeds are densely sampled and physically contacted with each other or when the illumination condition of seeds is inconsistent, these seeds cannot be effectively segmented into individual seed to calculate each individual seed phenotype parameters, and these tailored image segmentation algorithms which are based on classic image processing technology are sensitive to the texture of object and illumination conditions [20]. Above all, traditional image processing methods show weak robustness and poor generalization ability. Instance segmentation network based on deep learning can achieves effective segmentation by learning the deep features of the images to solve above problems [21, 22].

Deep learning has gathered a wide attraction from scientific as well as industrial communities [23]. In the field of computer vision. Convolutional Neural Networks (CNN) are widely applied in various tasks, such as classification [24, 25], object detection [26, 27], semantic segmentation and instance segmentation [28, 29], which greatly improves the results while traditional image processing methods can’t achieve [30]. With the rapid development of massively parallel Graphics Processing Unit (GPU) computing technology and big data processing technology, the widespread success of deep leaning techniques has spawned a multitude of applications in computer vision-based plant phenotyping [22], including weed detection [31], crop disease diagnosis [25], fruit detection [32] and many other applications listed in recent reviews [33, 34].

Deep learning applied in quantitative image analysis has grown exponentially in the past few years. However, training an accurate deep learning model with strong generalization ability requires a large amount of labeled data which is one of the disadvantages of deep learning. Compared with relatively common tasks (ImageNet classification [35] and COCO detection [36]), the need of annotated data for specialized tasks in agricultural applications is even more pronounced [21, 37, 38]. Although many techniques aiming to decrease the cost of expert labeling cost (such as domain adaptation [39] or active learning [21]) without compromising performance have been widely used in plant phenotyping fields, the annotations of phenotyping dataset is still necessary for algorithms evaluation, and the labelling process is tedium, painful, labor-intensive and time-consuming. Especially in the phenotyping of high-throughput crop seeds, the annotation of crop seed instance segmentation dataset will be a tremendous challenge.

An improvement to reduce the cost of manual annotation is learning from synthetic images. Although the synthetic image dataset is not authentic compared with real-word dataset, the important advantages of synthetic image dataset is that ground truth annotations can be automatically obtained without manual labor. Furthermore, the synthetic image approach equips with the ability of creating almost unlimited amount of labeled training dataset. Synthetic data can represent changes in a variety of conditions, which is usually difficult to achieve through image augmentation techniques on real sense images. Kuznichov et al. [40] proposed a method to segment and count the leaves of Arabidopsis, avocado and banana, by using synthetic leaf texture located with different sizes and angles to simulate images obtained in real agricultural scenes. Toda et al. [41] proved that synthetic datasets, which rendered the combination and direction of seeds, was sufficient to train an instance segmentation network to segment the high throughput barley seeds from real-world images. Collectively, synthetic image datasets have a great potential in computer vision-based plant phenotyping research field.

Transfer learning, which exploits the related knowledge in source domain to help the learning of the target domain [42], is one of the effective approaches which can reduce the costs of manual annotation and computing cost on the target domain dataset. Bosilj et al. [31] studied the role of deep learning-based knowledge transfer for different various of crop, with the purpose of reducing the training time and manual annotation work required in new task. The author proved that transfer learning could be used between different crops and could reduce training time by up to 80%. Coulibaly et al. [43] proposed a method of using transfer learning and feature extraction to realize the identification of pearl millet mildew, and achieved 95% accuracy, 94.5% recall and 91.75% F1-score. Sakurai et al. [39] investigated the effectiveness of transfer learning in plant segmentation tasks. In summary, transfer learning has great potential in the field of plant phenotyping, which can not only reduce the cost of data annotation, but also reduce the training time on new tasks.

To efficiently tackle individual soybean seed quick segmentation for high-throughput soybean seeds phenotype data extraction at individual seed level, we propose a method based on Mask R-CNN and transfer learning. Since the deep learning-based instance segmentation requires a large amount of labeled data, and the number of soybean seeds in each image is abundant, the labeling process is destined to be labor-intensive and time-consuming. Hence, we instead train with synthetic soybean seeds images dataset which were prepared by our novel synthetic image generation and augmentation approach which can generate the origin image and labeled image pair synchronously. The approach presented herein is motivated by high throughput soybean seeds phenotyping task. This work built on pioneer research on Mask R-CNN network, retrained by our synthetic labeled image dataset.

The paper’s contributions:

1. A method was proposed for rapidly and automatically generating synthetic labeled high throughput soybean seeds image dataset.
2. A hybrid sim/real dataset was designed for training and evaluating high throughput soybean seeds instance segmentation methods transferring from simulation to reality robustly.
3. A synthetic image dataset based Mask R-CNN with transfer learning was adapted to perform high throughput soybean seeds instance segmentation.
4. Multi-group comparation experiments were designed to evaluate the sim-to-real generalization abilities of Mask R-CNN trained by our synthetic dataset.

## Methods

### Raw soybean seeds image acquisition

**Fig. 1:**
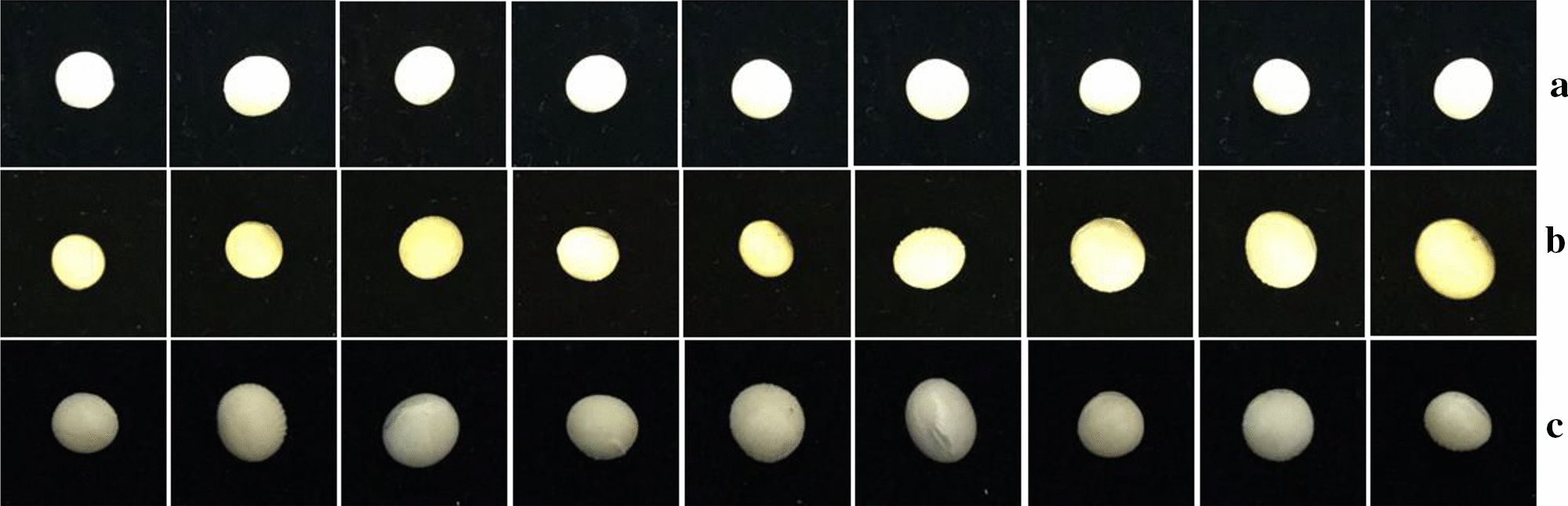
The soybean seed images were captured in three kind of illumination conditions. **a** outdoor scene in the daytime; **b** indoor scene in the daytime with fluorescent light; **c** indoor scene at night with fluorescent light

Soybean seeds used in this research were zhonghuang-30 and zhonghuang-42 which were supplied by Ministry of Agriculture and Rural Affairs Key Laboratory of Soybean Biology, the Institute of Crop Sciences, Chinese Academy of Agricultural Sciences. Zhonghuang-30 is an early-maturing variety of northern spring soybeans with a growth period of about 124 days. The plant height is about 64 cm. The number of main stem nodes is 15, and the effective branches are 1.1. Round leaves, purple flowers, brown hair, determinant growth habit. The grains are round, the seed coat is yellow, with a weak luster, brown hilum, and the weight of one hundred seeds is 18.1 g. Resistance to mosaic virus disease and gray spot disease. The fat content is 21.44% and the protein content is 39.53%. Zhonghuang-42 has an average growth period of 116 days. The plant height is about 71.1 cm, the effective branches are 0.9. The number of seeds per plant is 62.0. The grains are oval, the seed coat is yellow, with luster, light brown hilum and the weight of 100 seeds is 27.2 g. Oval leaves, purple flowers, gray hair, determinant growth habit. The average crude protein content is 45.08%, and the crude fat content is 19.23%. All the soybean seeds were threshed manually. The detail phenotypic descriptors of these varieties were summarized in Table 1. The single soybean seed upon the black-colored flannel was captured by the camera sensor of an iPhone 6 s plus (Apple) erected on a tripod with the image size of 3024 × 3024 at 72 dpi in three kind of illumination conditions as shown in Fig. 1. The working distance of camera sensor was fixed about 15 cm above the black-colored flannel background.

**Table 1:** The detail phenotypic descriptors of zhonghuang-30 and zhonghuang-42 soybean seeds

Soybean varieties	Seed shape	Seed coat color	Hilum color	100 grain weight
Zhonghuang-30	Round	Yellow	Brown	~ 18.1 g
Zhonghuang-42	Oval	Yellow	Light brown	~ 27.2 g

### Software libraries and hardware

The processing unit was a Lenovo Y7000P laptop with an Intel Core i7-9750H@2.60 Hz CPU, 16 GB RAM, and single GPU (Geforce GTX1660 Ti, NVIDIA). The environment of deep-learning-related procedure included Integration Develop Environment (IDE) integrating Python 3.6, Keras (ver. 2.1.5), Tensorflow_GPU (ver. 1.13.1) OpenCV3 (ver. 3.4.2), which were operated in Windows 10 64bit. The synthetic image-related procedure was operated on the same environment (GPU was not involved in computation). The manually annotation of real-world soybean seeds image was operated on the same environment using LabelMe (ver. 3.16.5).

### Synthetic image generation and augmentation

We randomly chose 200 soybean seeds for each cultivar (total of 400; 200 seeds for 2 cultivars), and each single soybean seed were placed above the black flannel and saved as an individual image file (total 400 seed images). These 400 seed images were used to create synthetic image datasets. The procedure of synthetic image generation was illustrated as following.

First, prepare a “background image pool (BIP)” and a “soybean seed image pool (SSIP)”. The BIP was prepared by capturing the actual black flannel background 10 times. The 10 background images were cropped at the fixed size of 256 × 256, 512 × 512, 1024 × 1024 randomly. The 10 different background images are different from each other as there are some dander of soybean seed on the black flannel. What needs to be pointed out is that the difference is not significant. And the SSIP was constituted by capturing a single soybean seed above the black flannel which made it convenient for the background subtraction.

Then, preprocess the image of the “soybean seed image pool”. Since the background was the black flannel, the classic threshold segmentation algorithm was opted to subtract the background. And it was cropped to get region of interest (RoI) as the soybean seed occupied a small area in the entire image, leaving a large blank space.

**Fig. 2:**
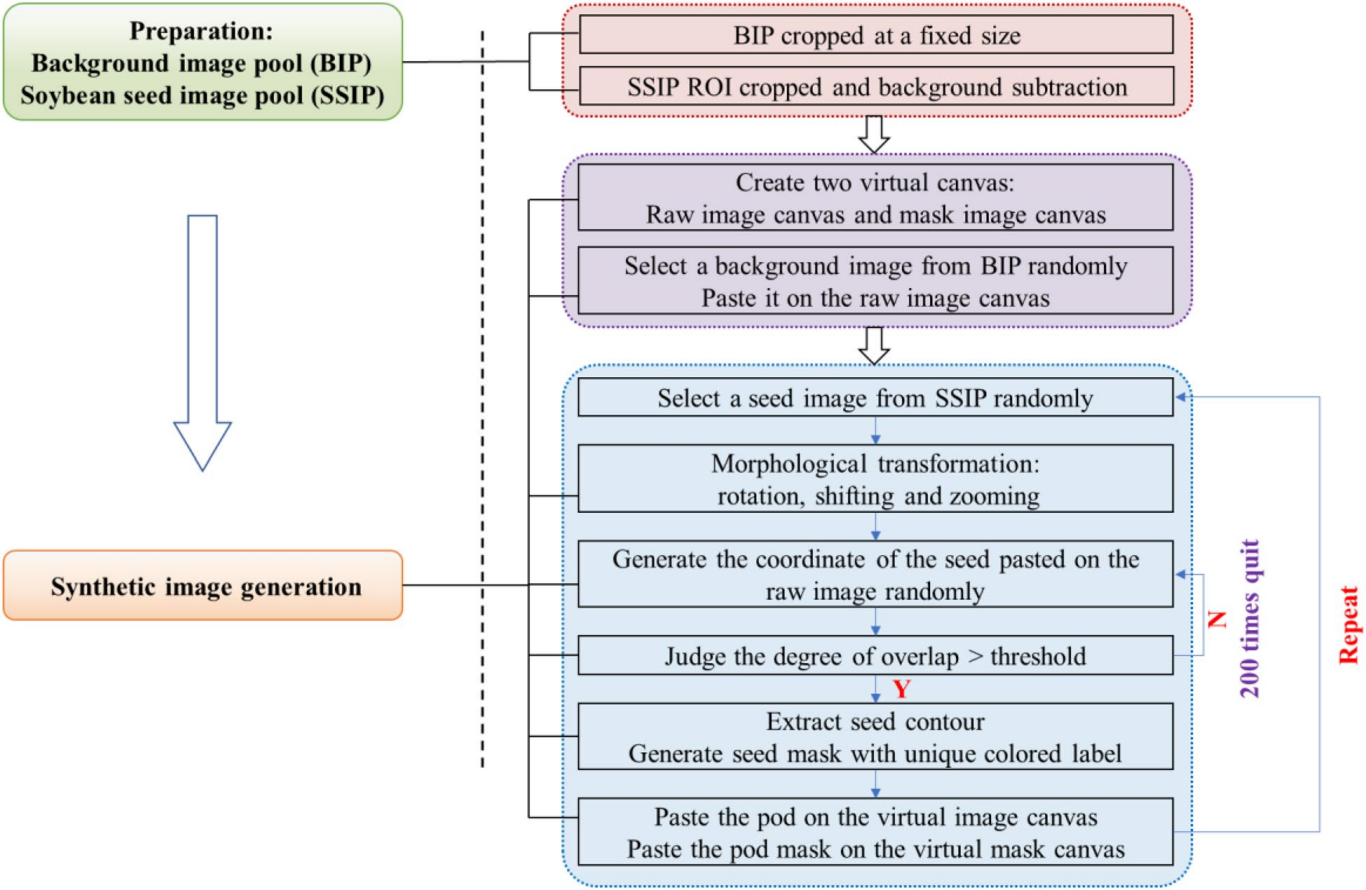
The procedure of one pair of synthetic images (raw image and mask image) generation and augmentation method

Last, synthesize high-throughput soybean seeds raw image and mask image pair. Firstly, select a background image from the BIP randomly and past it on the raw image canvas. Secondly, select a seed image randomly from the preprocessed SSIP and rotate and zoom the seed image randomly. Then, get the seed area and paste it on the coordinate $\left( {x,y} \right)$ of the raw image canvas. The coordinate $\left( {x_{i} ,y_{i} } \right)$ was randomly determined but restricted by the canvas size and the minimum Euclidean distance between the new coordination $\left( {x_{i} ,y_{i} } \right)$ and the coordinate $\left( {x_{j} ,y_{j} } \right)$ of the soybean seeds pasted on the canvas before to adjust the degree of overlap. The detailed restriction of the coordinate $\left( {x_{i} ,y_{i} } \right)$ was shown in the Formula 1. Thirdly, generating the corresponding mask image canvas by filling the seed area with different color selected from “Jet” colormap randomly and pasting the colored seed on the coordinate $\left( {x_{i} ,y_{i} } \right)$ of the mask image canvas with black background. After the above three steps, one soybean seed was labeled in one color automatedly. Lastly, repeat above three steps until the coordinate $\left( {x_{i} ,y_{i} } \right)$ can’t meet the minimum Eucliean distance requirments. Above all, a pair of synthetic high throughput soybean seeds raw image and mask image was generated which each single soybean seed in raw synthetic image was pasted on the corresponding position of mask image and was labeled in different color automatedly. Above all, the procedure of synthetic image datasets generation and augmentation method was shown in Fig. 2.

1 $$\begin{gathered} \left( {x_{i} ,y_{i} } \right) = \left\{ {\begin{array}{*{20}c} {random} & {i = 1} \\ {\min \sqrt {\left( {x_{i}^{center} - x_{j}^{center} } \right)^{2} - \left( {y_{i}^{center} - y_{j}^{center} } \right)^{2} } \ge thershold} & {i > 1} \\ \end{array} } \right. \hfill \\ \left( {0,0} \right) \le \left( {x_{i} ,y_{i} } \right) < \left( {canvas.shape(0),canvas.shape(1)} \right) \hfill \\ 1 \le j \le \left( {i - 1} \right) \hfill \\ \end{gathered}$$

$\left( {x_{i} ,y_{i} } \right)$ was the coordinate of the ith soybean seed pasted on the canvas; $\left( {x_{i}^{center} ,y_{i}^{center} } \right)$ was the center point coordinate of the i^th^ soybean seed pasted on the canvas; $\left( {x_{j}^{center} ,y_{j}^{center} } \right)$ was the center point coordinate of the 1st ~ (i-1)th soybean seed pasted on the canvas.

2 $$threshold = length \times radio$$

*Length* was the sum of diagonal distance of the bounding box of two soybean seeds, *ratio* is man-made parameter which used to control the overlapping of two soybean seeds.

As illustrated in Formula (2), the minimum threshold is related to the size of each soybean seed, which is a variable. Thus, we can adjust the ratio parameter manually to control the overlapping to obtain our desired synthetic results. For example, if we want to generate heavily overlapped image, we can decrease the ratio, otherwise vice versa. In this paper, the ratio is set 0.3.

### Real-world soybean seeds test dataset preparation

**Fig. 3:**
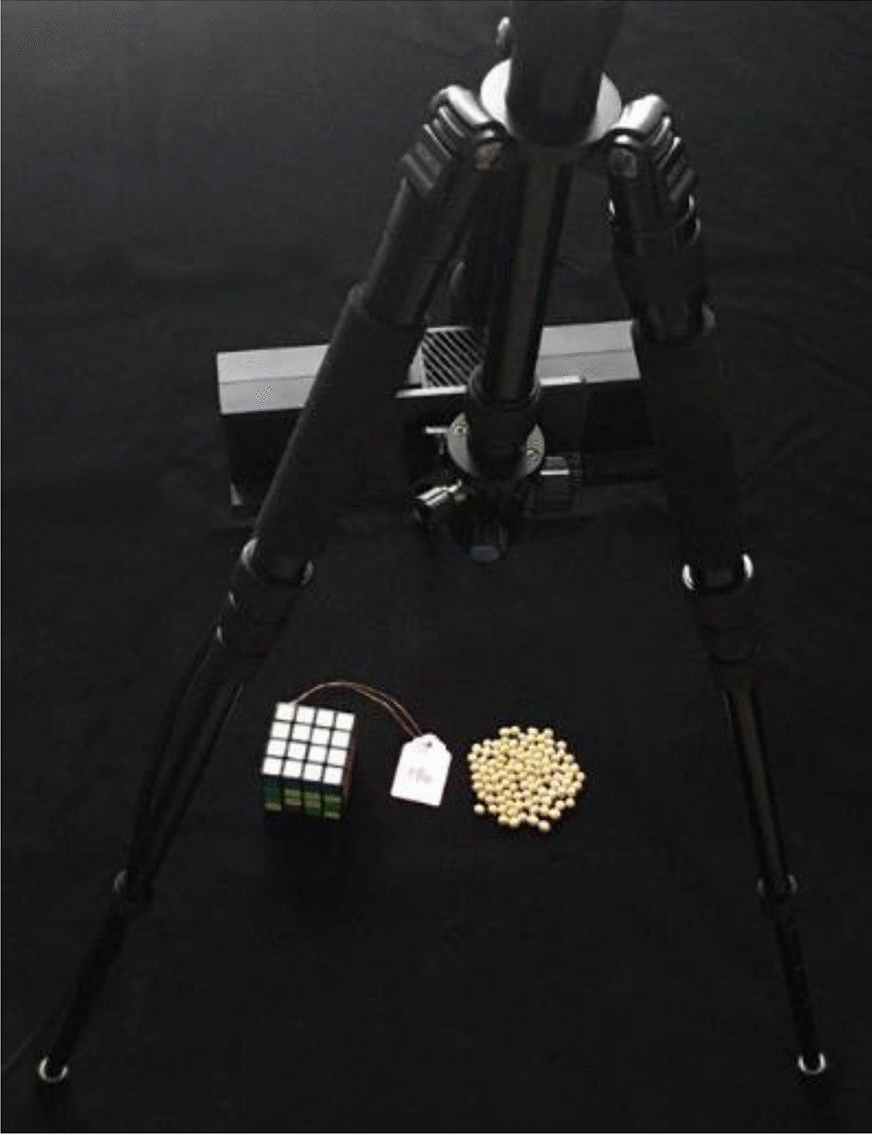
The acquisition scene setting of real-world high throughput soybean seeds

While we generated the synthetic soybean seeds test dataset by the method described in the previous section, a real-world soybean seeds test dataset was prepared consisting of 40 images by the following steps: (a) use a 100-seed board to select about 100 soybean seeds randomly one time; (b) tile these seeds upon the black-colored flannel randomly and make these seeds densely sampled (e.g., physically touching) to simulate the phenotypic investigation in the real scene; (c) capture 8 images (4 images for 2 cultivars) with the image size of 3024 × 3024 by the camera sensor of an iPhone 6 s plus (Apple) erected on a tripod with about 0.3 m working distance and 32 images (16 images for 2 cultivars) with the image size of 1920 × 1080 at 96 dpi by the RGB sensor of Kinect v2 (Microsoft, Redmond, WA, USA) erected on a tripod with about 0.75 m [20] working distance as shown in Fig. 3. The detail of real-world soybean seeds test dataset preparation was summarized in Table 2. Before manual annotation, the images were cropped according to the region of interest (RoI). The real-world soybean seeds images with the seeds heavily and physically touching, which were annotated by LabelMe [44] manually, were used as testing dataset for assessing the generalization ability of the model retrained by our synthetic datasets. The manual annotation results were explained in the later section.

**Table 2:** The detail information of our real-world soybean seeds test dataset

Test image	Number of images	Image size	Seed counts	Sensor	Imaging distance
Test dataset_1	8	3024 × 3024	~ 100	iPhone 6 s plus	~ 0.3 m
Test dataset_2	32	1920 × 1080	~ 100	Kinect v2	~ 0.75 m

### Model training

**Fig. 4:**
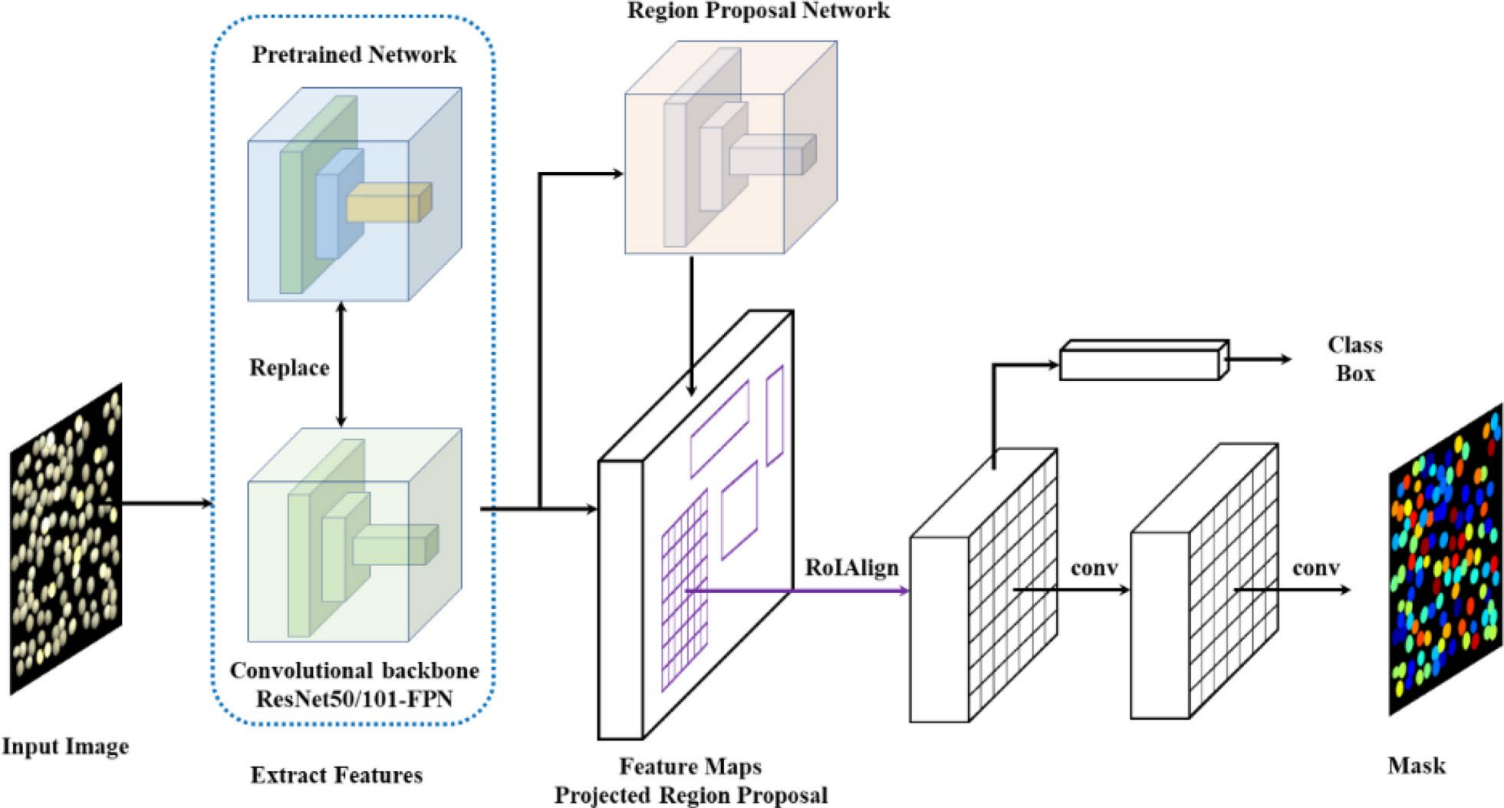
Mask R-CNN with the pretrained network for high-throughput soybean seeds instance segmentation

Mask R-CNN [45], consolidated by an object detection algorithm Faster R-CNN [46] and a semantic segmentation algorithm fully convolution network (FCN) [47] as shown in Fig. 4, is a sophisticated segmentation method, which can be trained by massive hand-labeled images datasets to segment specific categories of object. A Mask R-CNN implementation on the Keras/Tensroflow backend [48] was opt after experimenting with various implementation. Two feature extraction architectures (ResNet50/101-FPN [49] backbone) were evaluated. Left–right, up-down, rotation, brightness and Gaussian blur image augmentations were used herein to increase the diversity of dataset. The batch size was 2 when the image size was 256 × 256 and 512 × 512, and was 1 when the image size was 1024 × 1024. Since we focus on training the mask branch, the loss weight of the mask was set to 2.0, the other loss weights are set 1.0. A connection dropout probability of 0.5 was added to the fully connected layers to prevent from overfitting. Table 3 was the network configuration which was selected empirically after training and analyzing the test results.

**Table 3:** Network parameters for high-throughput soybean seeds instance segmentation using Mask R-CNN

Parameters	Values
Backbone layer	ResNet 50/101
Head layer	Faster R-CNN
Input size	256*256 / 512*512/1024*1024
Anchor ratio	[0.5, 1, 2]
Learning rate	0.001
Epoch	40
RPN anchor scale	(8, 6, 32, 64-128)
Pre-processing	Mean-subtraction
Image resize mode	None
Augmentation	LR, UD, Rotation, Brightness, Gaussian Blur

Before the model training, two pre-trained model weights based on MS-COCO dataset [36] and synthetic barley dataset [41], were introduced using transfer learning to solve the problem of high-throughput soybean seeds instance segmentation. Retraining on the basis of the pre-trained model was divided into two steps: (1) Only train the head layers which include the RPN, classifier and mask heads of the Mask R-CNN. And the weights of the heads are randomly initialized by default xavier initializer and zeros bias initializer. In order not to weaken the feature extraction ability of the backbone layer, we frozen all backbone layers and only trained the randomly initialized head layers for 20 epochs. (2) Fine-tune all layers. To better adapt on our new dataset, we fine-tuned all layers for 20 epochs after training the head layers. The reason why 20 epochs are considered will be illustrated in experiments and results section.

### Model evaluation metrics

To evaluate the accuracy of high-throughput soybean seed instance segmentation model, two indicators included average precision (AP) and recall, used to evaluate in the original research [44], were also used herein.

The result of a model prediction is classified as true positive (TP), false positive (FP), true negative (TN), false negative (FN). The precision and recall are calculated by the following Formula 3:

3 $$\begin{gathered} Precision = \frac{TP}{{TP + FP}} \hfill \\ Recall = \frac{TP}{{TP + FN}} \hfill \\ \end{gathered}$$

Intersection over union (IoU) is a basic evaluation indicator and it measures the overlap of two regions, which is the ratio of the overlap of the two regions to the total area of the two (the overlap is only calculated once) as shown below:

4 $$IoU = \frac{area \, of \, overlap}{{area \, of \, union}}$$

To calculate the values of Recall, we use bounding boxes IoU. For each ground-truth bounding box, when the detected bounding box overlaps the ground-truth over the IoU threshold, we considered it was the correct detection, which was counted as TP, otherwise we considered it was the wrong detection (FP). And when the predicted bounding box with no ground-truth, we determined it was FN.

AP is defined as the area under the curves (AUC) of precision and recall using different confidence of the detected soybean seed. And it is evaluated at 10 different masks IoU threshold levels from 0.5 to 0.95 with the interval of 0.05. AP_50_ and AP_75_ are the prediction accuracy rates when the masks IoU threshold are 0.5 and 0.75, respectively. As AP_75_ requires correct matching with more precise masks, AP_75_ is more stringent than AP_50_. AP@ [0.5:0.95] is the average value of APs with all the masks IoU thresholds.

**Fig. 5:**
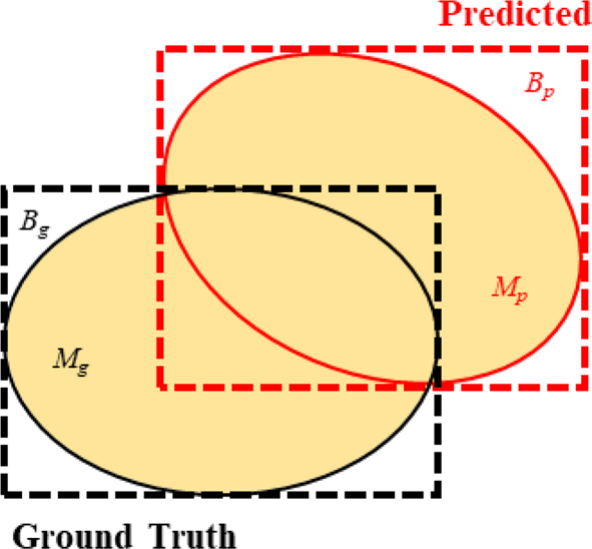
The definition and principles of the bounding boxes IoU and masks IoU. Where B_g_ is ground truth bounding boxes, M_g_ is the ground truth masks, B_p_ is the predicted bounding boxes, M_p_ is the predicted masks

The definition and principles of the bounding boxes IoU and masks IoU were depicted in Fig. 5. And they were calculated by the following equation:

5 $$\begin{gathered} IoU = \frac{{B_{g} \cap B_{p} }}{{B_{g} \cup B_{p} }} \hfill \\ Mask IoU = \frac{{M_{g} \cap M_{p} }}{{M_{g} \cup M_{p} }} \hfill \\ \end{gathered}$$

### Qualification of soybean seed morphology

After high-throughput soybean seeds were segmented into individual single seed, the seed morphology phenotype quantification was applied. We use the “measure.regionprops” module of the scikit-image library to calculate the morphological parameter of the seed, such as length and width.

**Fig. 6:**
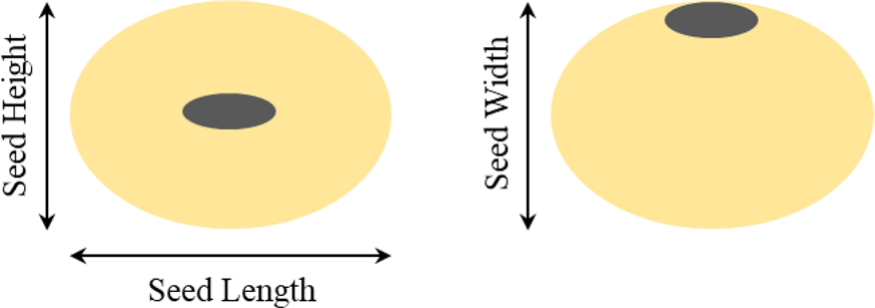
seed length, width and height of soybean seed

The soybean seed shape traits are defined in the Fig. 6. In our study, as the high-throughput soybean seeds phenotype analysis was based on two-dimensional image, it was impossible to obtain the seed length, seed height and seed width of soybean seed from one image synchronously. Hence, we considered a hypothesis that average value of the seed height and seed width measured by a digital vernier caliper is the reference of seed width in our study.

We select 100 soybean seeds for each cultivar with a 100-seed board randomly, and use a digital vernier caliper to measure each seed length height and width three times, and then calculate the average value as the seed shape phenotype data of this soybean seed.

## Experiments and results

### Preparation of soybean seeds dataset

**Fig. 7:**
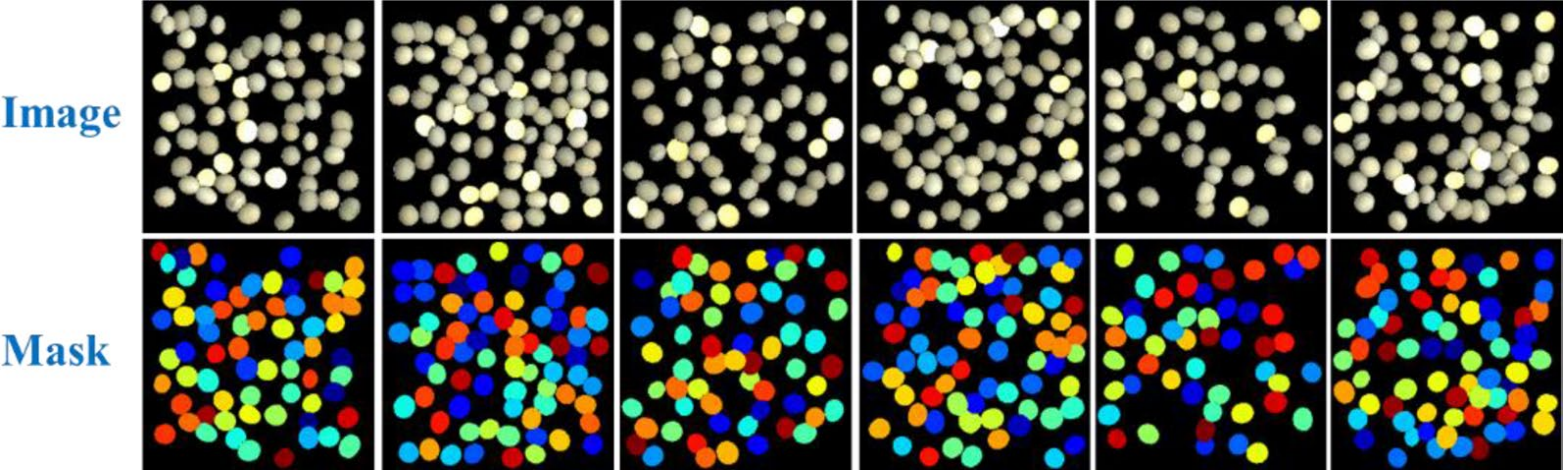
Some examples of labeled synthetic images. The first row shows the synthetic raw images, and the second row shows the corresponding labeled mask images

We generated images with size of 256*256, 512*512, 1024*1024 respectively, and the soybean seeds were randomly located inside the canvas region by our procedure as shown in Fig. 7. We prepared a small training dataset and a large training dataset for each size of synthetic image to fine-tune the pretrained Mask R-CNN. The small training dataset constituted by 220 set of image pairs of synthetic soybean seeds images and its mask images, 200 of those images for training, 20 for validation. And 1100 set of image pairs constituted the large training dataset, 1000 for training, 100 for validation. We also prepared another new 200 set of image pairs for each image size as synthetic test dataset, and these synthetic images were not used in the model training or validation. The generation time was about 274, 487, 575 min respectively for all the datasets of each image size. The preparation of synthetic image datasets of soybean seeds was shown in Table 4.

**Table 4:** The detail of three different image size of 256*256, 512*512, 1024*1024 datasets for training, validation and testing generated by our image synthetic method

Image size	Seed count	Large dataset	Small dataset	Test dataset	Generation time/min
		Train./Val.	Train./Val.		
256*256	50–80	1000/100	200/20	200	274
512*512	80–100	1000/100	200/20	200	487
1024*1024	100–120	1000/100	200/20	200	575

**Fig. 8:**
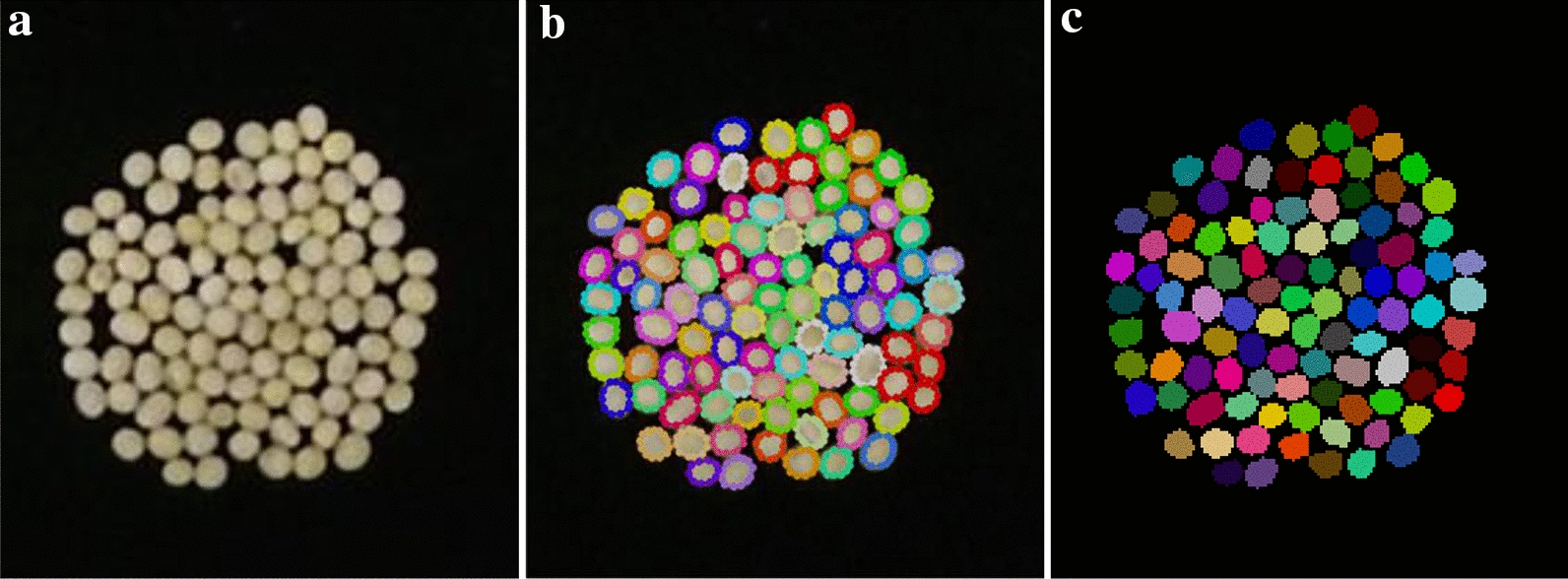
One labeled image data of real-world soybean seed test dataset. **a** raw image, **b** annotated by LabelMe, **c** Instance masks

In the preparation of the real-world soybean seeds test dataset, a sample image of real-world soybean seeds test dataset as shown in Fig. 8, the time of manual annotation process with LabelMe was about 60 min per image. Compared with the preparation of real-world soybean seeds test dataset, which had a plethora of soybean seeds per image and the labor-intensive annotation process of the test dataset was destined to be extremely tedious, our synthetic image generation and augmentation method can prepare plenty of labeled image dataset according to our experiments and can decrease the labor cost significantly. In addition, the real-world soybean seeds image dataset labeled by LabelMe with the contour of soybean seed was fitted by a polygon as shown in Fig. 8(b) which we tried our best to better fit the soybean seeds contours. From Fig. 8(c), we can obviously distinguish that the manually labeled real-world soybean seed image was not better than our synthetic labeled image, for the contours of soybean seeds fitted by polygons were not smooth resulting the instance masks of soybean seeds were not real.

### Instance segmentation results of soybean seed

**Fig. 9:**
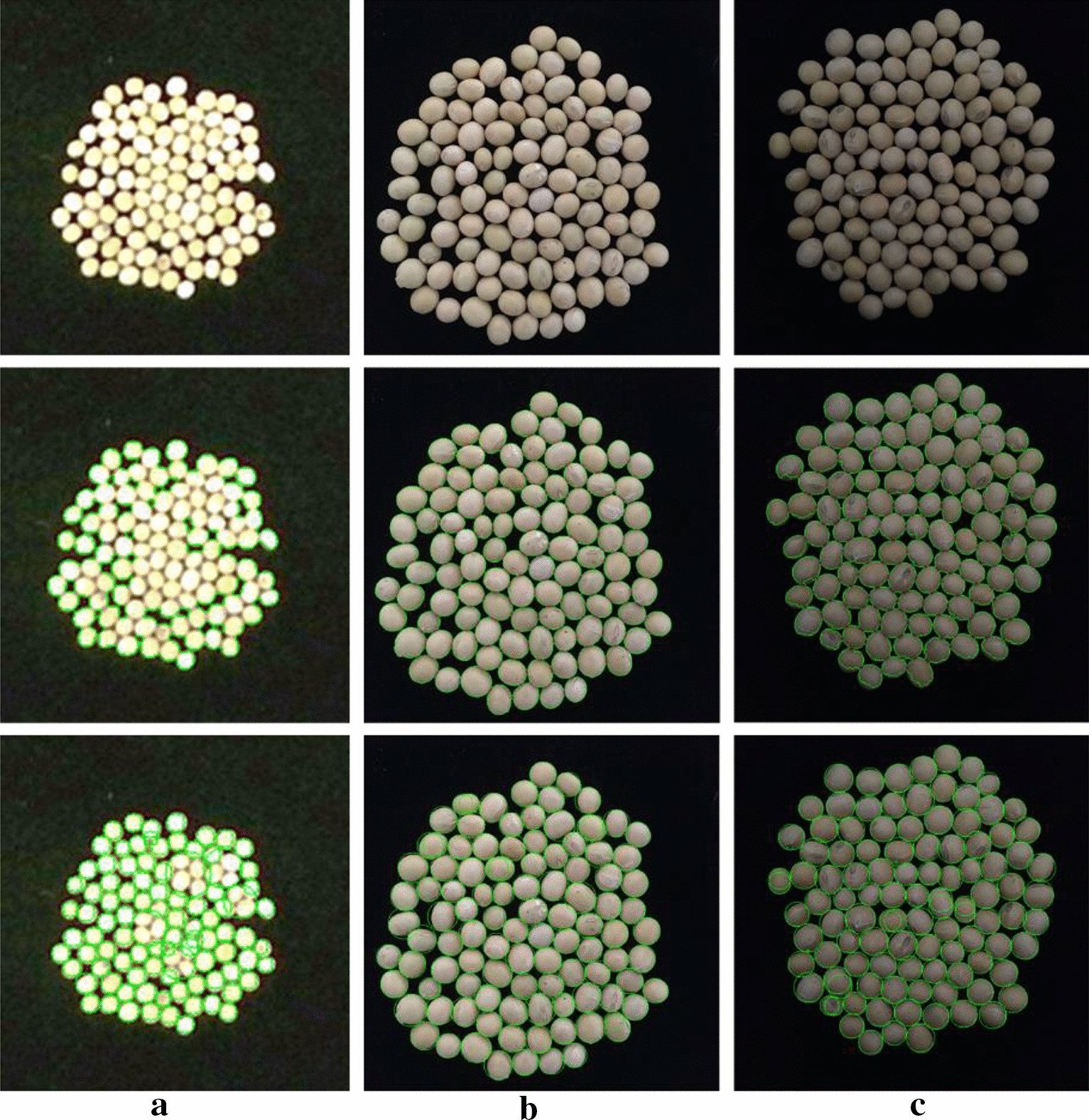
An example of the visual results using unsupervised segmentation methods on our real-world soybean seed images. **a** real-world soybean seeds test image captured by Kinect v2 outdoor, **b** real-world soybean seeds test image captured by iPhone 6 s plus at indoor scene in the daytime with fluorescent light, **c** real-world soybean seeds test image captured by iPhone 6 s plus at indoor scene at night with fluorescent light. The first row was the input soybean seeds test image, the second row was the results of the thresholding and contour extraction method, and the third row was the results of watershed algorithm

Before exhibiting the results of object detection and instance segmentation with Mask R-CNN, we used two unsupervised segmentation methods like contour detection methods and watershed algorithm on our real-world soybeans test dataset. However, both of them failed to segment the soybean seeds which were heavily overlapping. As illustrated in the second row of Fig. 9, we employed basic thresholding and contour extraction approach to identify the contour of soybean. The result showed that a group of soybean seeds are in one contour, and in fact those seeds are multiple, which was an inaccuracy segmentation. Comparing to thresholding and contour extraction method, watershed algorithm performed better as shown in the third row of Fig. 9, but it also failed to extract all objects when target objects overlap or touch densely with each other. Additionally, unsupervised method depends on empirical parameter, which is fussy to tune the parameters to obtain satisfying result, furthermore the parameter varies with different target object layout.

**Fig. 10:**
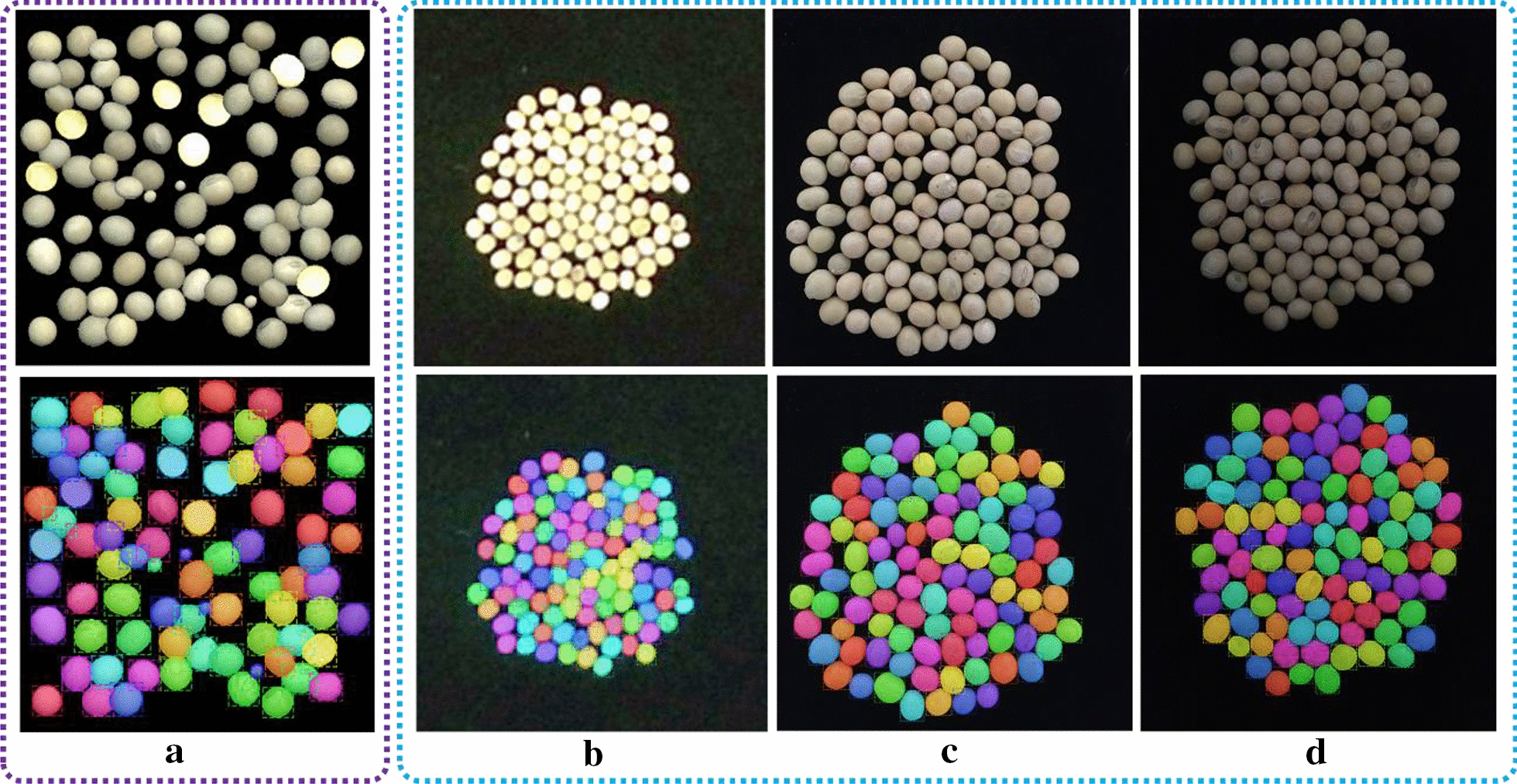
One example of visualized results of output of synthetic soybean seeds test image and real-world soybean seeds test image. **a** synthetic soybean seeds test image, **b** real-world soybean seeds test image captured by Kinect v2 outdoor, **c** real-world soybean seeds test image captured by iPhone 6 s plus at indoor scene in the daytime with fluorescent light, **d** real-world soybean seeds test image captured by iPhone 6 s plus at indoor scene at night with fluorescent light. The first row was the input soybean seeds test image, the second row was the results of our model output

The visual results and the quantitative of evaluation metrics of object detection and instance segmentation with Mask R-CNN were illustrated herein. The output of the trained Mask R-CNN model was a set of classes, bounding boxes coordinates and masks images of soybean seed regions. One example of visualized results of synthetic soybean seeds test image and real-world soybean seeds test images in different illumination conditions with different imaging sensors was shown in Fig. 10, which showed that the soybean seeds were accurately located and segmented by the trained model regardless of their shape, size, location, illumination condition and resolution.

Above, traditional unsupervised methods rely on texture features of object, and it is also sensitive to the layout of target objects and illumination, which bring about instability. Whereas machine learning-base method training a model by quantity of data, it solves the shortage of traditional methods in aspect of empirical based parameter tuning and instability.

The model was also evaluated by the test datasets which included synthetic image dataset with three kind of image size and real-world soybean seed image dataset. Tables 5 and 6 summarized the quantitation of evaluation metrics of the model retrained by our large dataset and small dataset of 256 × 256 px with COCO weights respectively. We can conclude that the ResNet101-FPN backbone layer can learn more features than ResNet50-FPN backbone layer particularly in small dataset. For a new instance segmentation task, comparing the real-world soybean seeds segmentation results, we came to the conclusion that the network with ResNet101-FPN trained by the large training dataset with 1000 images brought the best expected gains, then the performance from high to low was the network with ResNet101-FPN trained by the small training dataset, the network with ResNet50-FPN trained by the large training dataset, the network with ResNet50-FPN trained by the small training dataset.

**Table 5:** The quantitation of evaluation metrics of the model retrained by the large dataset of 256 × 256 px with COCO weights

Dataset	Large dataset in the image size of 256 × 256							
Pre-trained model	Pre-trained COCO weights [35]							
Backbone layer	ResNet50-FPN				ResNet101-FPN			
Test dataset	Synthetic			Real-world	Synthetic			Real-world
	256	512	1024		256	512	1024	
Recall_50_	0.99	0.99	0.97	0.86	0.99	1.0	0.97	1.0
AP_50_	0.99	0.99	0.98	0.83	0.99	1.0	0.98	1.0
AP_75_	0.99	0.93	0.95	0.64	0.99	1.0	0.98	0.98
AP@[0.5:0.95]	0.78	0.68	0.65	0.50	0.90	0.85	0.82	0.72

**Table 6:** The quantitative of evaluation metrics of the model retrained by small dataset of 256 × 256 image size with COCO weights

Dataset	Small dataset in the image size of 256 × 256							
Pre-trained model	Pre-trained COCO weights [35]							
Backbone layer	ResNet50-FPN				ResNet101-FPN			
Test dataset	Synthetic			Real-world	Synthetic			Real-world
	256	512	1024		256	512	1024	
Recall_50_	0.75	0.84	0.79	0.45	0.88	0.65	0.57	0.91
AP_50_	0.76	0.85	0.82	0.54	0.92	0.65	0.62	0.91
AP_75_	0.76	0.58	0.40	0.11	0.92	0.64	0.62	0.89
AP@[0.5:0.95]	0.67	0.51	0.43	0.22	0.80	0.50	0.47	0.66

Tables 7 and 8 summarized the quantitation of evaluation metrics of the model retrained by our large dataset and small dataset of 256 × 256 px with synthetic barley weights respectively. Same as retrained by COCO weights, the ResNet101-FPN backbone layer can learn more features than ResNet50-FPN backbone layer particularly in small dataset.

**Table 7:** The quantitation of evaluation metrics of the model retrained by the large dataset of 256 × 256 px with synthetic barley weights

Dataset	Large dataset in the image size of 256 × 256							
Pre-trained model	Pre-trained barley weights [40]							
Backbone layer	ResNet50-FPN				ResNet101-FPN			
Test dataset	Synthetic			Real-world	Synthetic			Real-world
	256	512	1024		256	512	1024	
Recall_50_	0.99	0.98	0.93	0.98	0.99	1.0	0.97	1.0
AP_50_	0.99	0.98	0.94	0.89	0.99	1.0	0.97	1.0
AP_75_	0.97	0.97	0.82	0.56	0.99	1.0	0.97	0.97
AP@[0.5:0.95]	0.79	0.72	0.62	0.50	0.92	0.86	0.82	0.71

**Table 8:** The quantitation of evaluation metrics of the model retrained by the small dataset of 256 × 256 px with synthetic barley weights

Dataset	Small dataset in the image size of 256 × 256							
Pre-trained model	Pre-trained barley weights [40]							
Backbone layer	ResNet50-FPN				ResNet101-FPN			
Test dataset	Synthetic			Real-world	Synthetic			Real-world
	256	512	1024		256	512	1024	
Recall_50_	0.30	0.05	0.10	0.02	0.99	1.0	0.88	1.0
AP_50_	0.33	0.05	0.12	0.01	0.99	1.0	0.93	1.0
AP_75_	0.32	0.04	0.06	0.00	0.99	1.0	0.93	0.99
AP@[0.5:0.95]	0.24	0.03	0.07	0.00	0.90	0.83	0.77	0.72

To compare the results of different pre-trained weights, we list a sample of comparative experiment retrained by our small training dataset of 256 × 256 image size by Mask R-CNN with ResNet101-FPN backbone as shown in Tables 6 and 8. The pre-trained weights included the COCO weights and the synthetic barley weights which was trained by a synthetic barley image dataset and similar to our high throughput soybean seeds instance segmentation task. Nevertheless, the generation ability of the synthetic barley model on our dataset was poor, where Recall_50_ = 0.016, AP@[0.5:0.95] = 0.055 on our synthetic soybean test dataset, and Recall_50_ = 0, AP@[0.5:0.95] = 0 on our real-world soybean test dataset. Comparing the results in Tables 5, 6 and 8, we found that finetune the synthetic barley weights with small training dataset can receive excellent results compared with COCO weights retrained by small training dataset and compared with COCO weights retrained by large training dataset. Thus, we can conclude that finetune a pre-trained model which was similar to our instance segmentation task with the small training dataset of 256 × 256 image size can achieve an excellent performance.

**Fig. 11:**
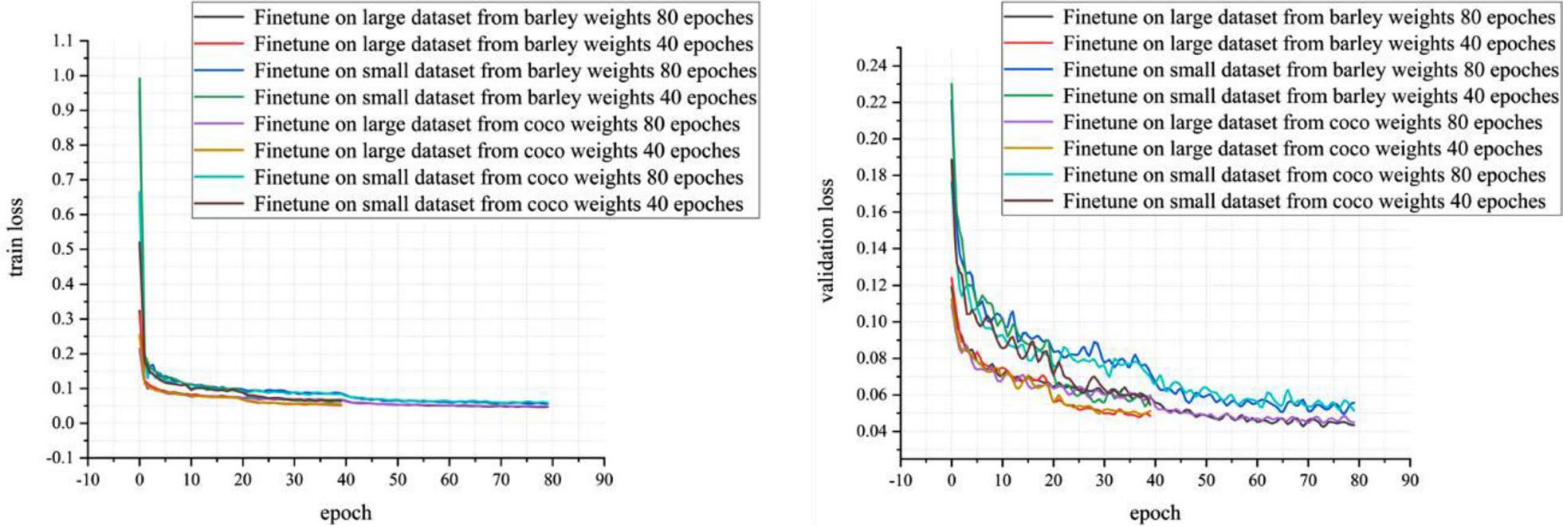
Loss curves of several different instance segmentation model in training stage: **a** training loss curves, **b** validation loss curves, which the training datasets were our synthetic datasets of 256 × 256 px and the pre-trained models included synthetic barley weights and COCO weights

The training loss and validation loss curves of the model with backbone layer ResNet101-FPN finetuned by two different training strategies were shown in Fig. 11. The two different training strategies, one was 20 epochs of head layers and 20 epochs of whole model and the other was 40 epochs of head layers and 40 epochs of whole model. The training datasets included our synthetic lager training dataset and the synthetic small training dataset of 256 × 256 px and the pre-trained models included synthetic barley weights and COCO weights. By learning the validation loss curves for two stages of fine-tuning, we found that about 20 epochs in the first stage is the inflection point. Hence, 20 epochs in the first stage were considered. Same as the second stage.

**Fig. 12:**
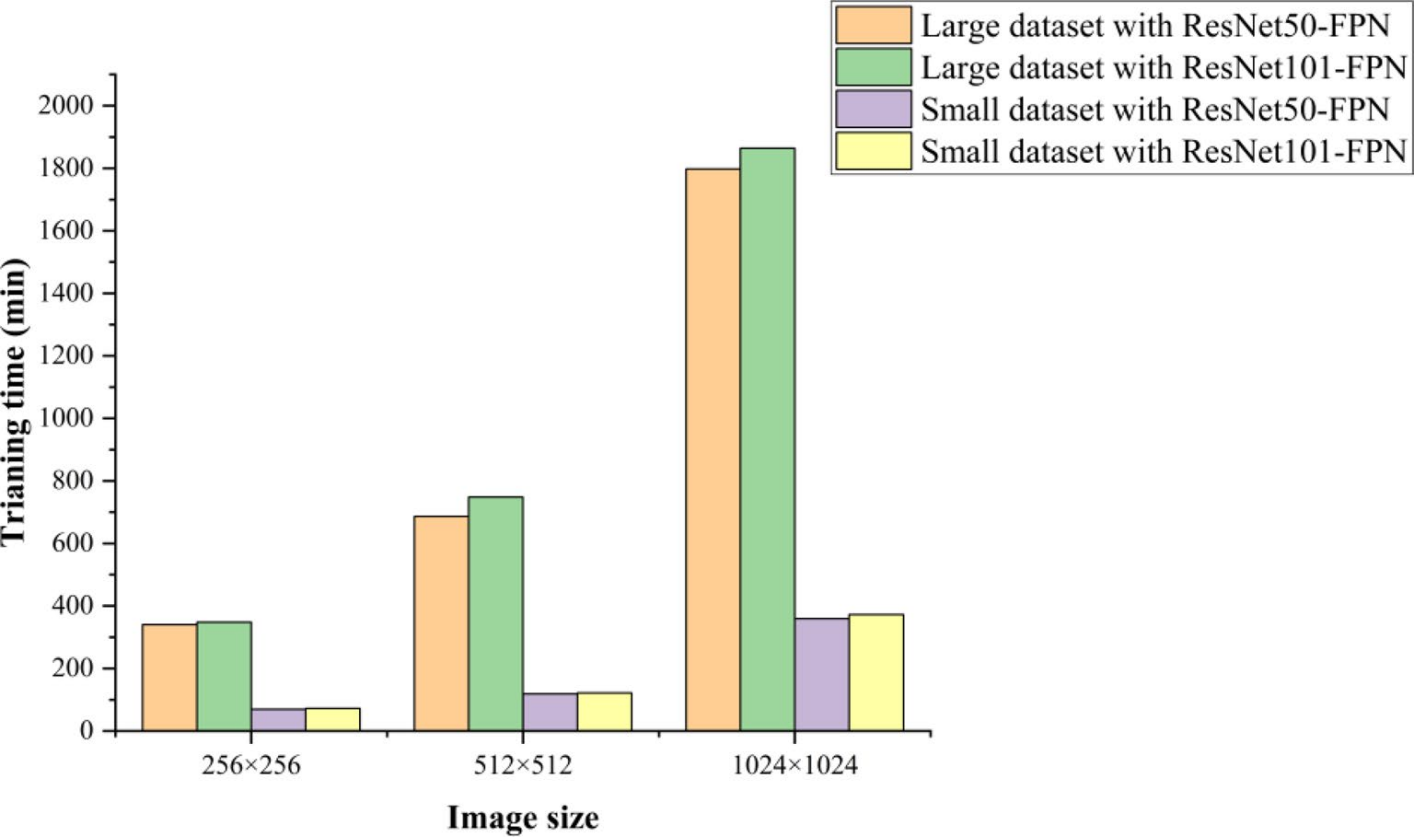
The training time with large dataset and small dataset in the image size of 256 × 256, 512 × 512, 1024 × 1024

At last, we also compared the model retrained by different image size of 256 × 256, 512 × 512, 1024 × 1024, and we found that the improved performance was not pronounced with increasing the image size as the texture of soybean seeds was simple. In addition, the training time with large dataset and small dataset in different image size of 256 × 256, 512 × 512, 1024 × 1024 was summarized in Fig. 12. It showed that the training time increased as the image size increasing, and the training time of large datasets was significantly longer than that of small datasets. Hence, we can conclude that it wasn’t indispensable to prepare a higher resolution training image dataset for instance segmentation with Mask R-CNN in our study and the Mask R-CNN network retrained by large dataset based on pre-trained COCO weights can be replaced by a small dataset based on the pre-trained synthetic barley weights, which similar to our high throughput soybean seeds instance segmentation task, but performed poor on our datasets, where Recall50 = 0.016, AP@[0.5:0.95] = 0.055 on our synthetic soybean test dataset, and Recall50 = 0, AP@[0.5:0.95] = 0 on our real-world soybean test dataset.

### Accuracy of length, width of each soybean seed

**Fig. 13:**
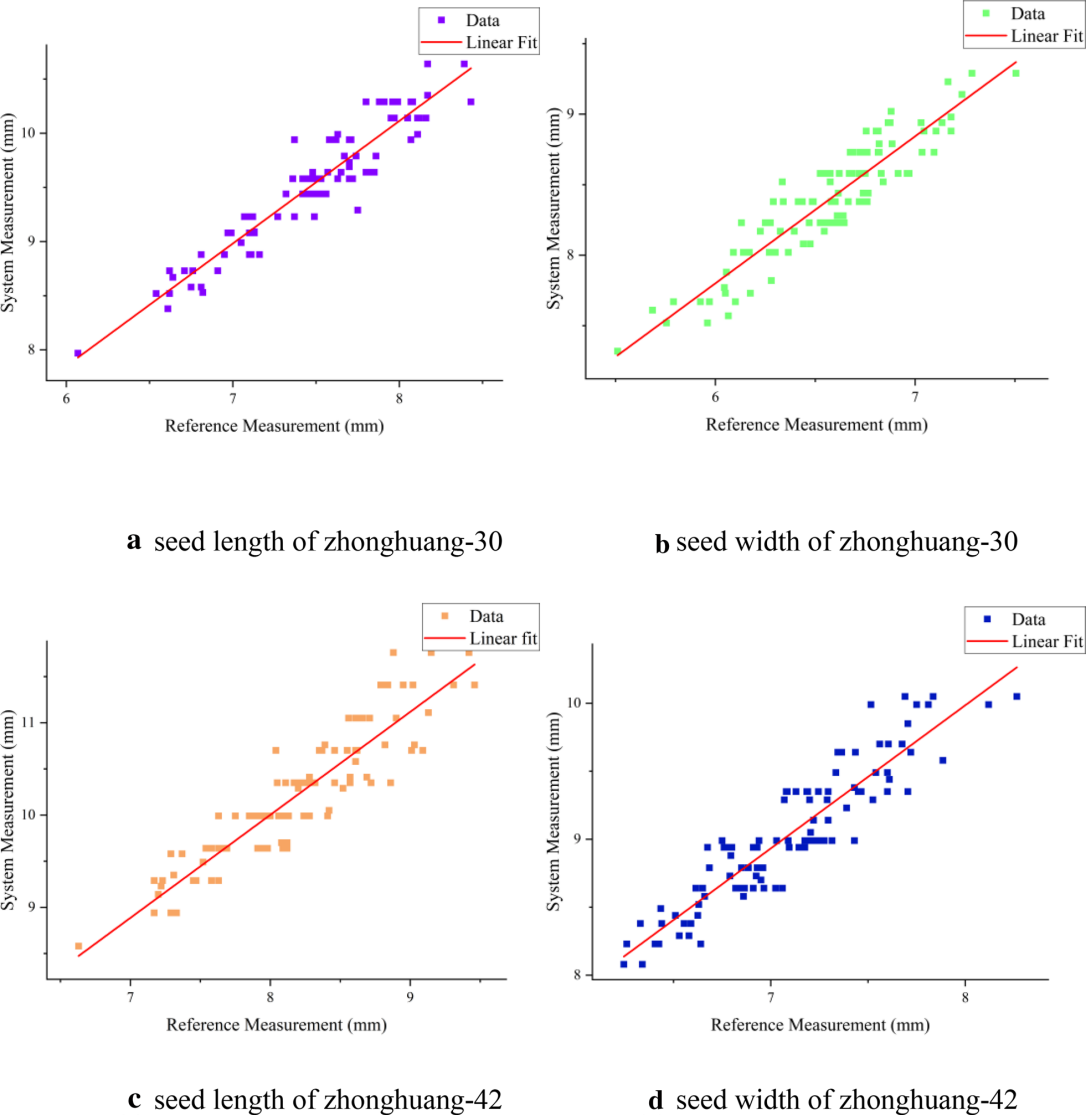
Data distribution and fitting results of (**a**) seed length (**b**) seed width of zhonghuang-30 and (**c**) seed length (**d**) seed width of zhonghuang-42

The results of soybean seed length and width measurement for zhonghuang-30 and zhonghuang-42 samples were shown in Fig. 13. The results showed that the system measurements, seed length (R^2^ = 89.26%) and width (R^2^ = 84.69%) of zhonghuang-30 and seed length (R^2^ = 88.11%) and width (R^2^ = 83.91%) of zhonghuang-42, had a fine linear relationship with the reference data. The average measurement error and the average relative error of the zhonghuang-30 and zhonghuang-42 were shown in Table 9.

**Table 9:** The average measurement error and the average relative error of the zhonghuang-30 and zhonghuang-42

Soybean varieties	Average measurement error		Variance of measurement error			Average relative error		Variance of relative error		
	Seed length/mm	Seed width/mm	Seed length	Seed width		Seed length/%	Seed width/%	Seed length		Seed width
Zhonghuang-30	2.04	1.82	0.04		0.03	27.4	27.9	6.63	8.33	
Zhonghuang-42	2.03	1.94	0.09		0.04	24.76	27.38	13.39	9.11	

**Fig. 14:**
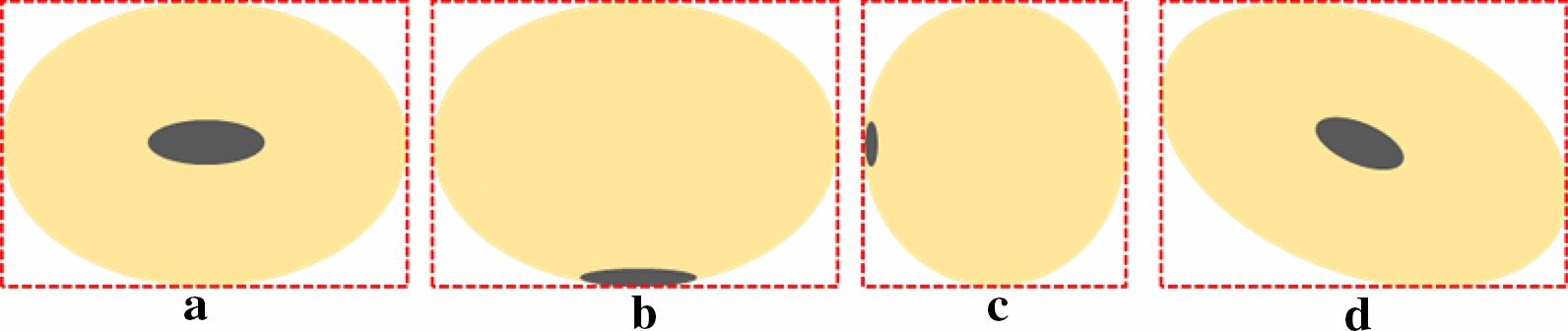
Examples of measurement errors which were introduced by different view angles and orientation of soybean seed

The reasons for the measurement errors were summarized as follows: (1) Errors were introduced by manual measurement. We need to manually take out one seed from the high-throughput soybean seeds in the corresponding position one by one and measure the seed length, width and height of the seed with a Vernier caliper, which was prone to error. (2) Errors were introduced by view angle of measurement. The view angle of manual measurement was not the view angle of camera, leading the manual measurement was inconsistent with the system measurement. The standard view angle of measuring seed length and seed width was shown in Fig. 14 (a). When the view angle looks like Fig. 14(b, c), the predicted seed width would greater than or less than the reference data which depended on the seed thickness. (3) Errors were introduced by our measurement approach. The bounding box-based instance segmentation method led to incomplete edges of the segmented instances, which in turn led to low accuracy of the obtained soybean seed morphological parameters. Pixel-based segmentation can be alternative to improve the performance of morphological parameter study [50]. And the soybean seeds were randomly orientated above the black flannel, however the bounding box of Mask R-CNN output didn’t consider the orientation of the segmented instances, which would also cause errors in the system measurement as shown in Fig. 14(d).

## Conclusion

The major contribution and advantages of our method are: (1) proposed a novel synthetic image generation and augmentation method working for preparing plenty of labeled image dataset for instance segmentation automatically which can pronouncedly decrease the labor cost of manual annotation. (2) The proposed transfer learning method by finetune the pre-trained model weights can reduce the computing costs significantly. (3) The pipeline proposed in our research can be expanded to the other high-throughput objects instance segmentation and morphology measurement.

However, our approach has a few limitations. Firstly, the high-throughput soybean seeds phenotype analysis was based on two-dimensional image which lacked depth information, it was impossible to obtain the seed length, seed height and seed width of soybean seed from one view-point image synchronously. Secondly, the computing cost of training the instance segmentation model is relatively high which still need to be improved. Lastly, our synthetic image generation and augmentation method is limited to one class object which need to be extended to synthetize more than one class object for multi-class objects instance segmentation.

In the future research, we intend to further improve the segmentation precision by pixel-based segmentation method and decrease the computing cost for the instance segmentation of high-throughput soybean seeds which are physically touching densely. And other datasets types like RGB-D dataset which can acquire more phenotype information by just retraining the instance segmentation network is left to the future work.

## Data Availability

The datasets and materials will be provided on publication.
